# A Review on the Impact of Oxidative Stress and Medicinal Plants on Leydig Cells

**DOI:** 10.3390/antiox12081559

**Published:** 2023-08-04

**Authors:** Elizabeth Monageng, Ugochukwu Offor, Ndivhuho Beauty Takalani, Kutullo Mohlala, Chinyerum Sylvia Opuwari

**Affiliations:** 1 Department of Medical Biosciences, Faculty of Natural Science, University of Western Cape, Cape Town 7535, South Africa; 2 School of Anatomical Sciences, Faculty of Health Sciences, University of the Witwatersrand, Johannesburg 2193, South Africa

**Keywords:** oxidative stress, reactive oxygen species, medicinal plants, Leydig cells, antioxidants

## Abstract

Leydig cells are essential for steroidogenesis and spermatogenesis. An imbalance in the production of reactive oxygen species (ROS) and the cellular antioxidant level brings about oxidative stress. Oxidative stress (OS) results in the dysfunction of Leydig cells, thereby impairing steroidogenesis, spermatogenesis, and ultimately, male infertility. To prevent Leydig cells from oxidative insults, there needs to be a balance between the ROS production and the cellular protective capacity of antioxidants. Evidence indicates that medicinal plants could improve Leydig cell function at specific concentrations under basal or OS conditions. The increased usage of medicinal plants has been considered a possible alternative treatment for male infertility. This review aims to provide an overview of the impact of oxidative stress on Leydig cells as well as the effects of various medicinal plant extracts on TM3 Leydig cells. The medicinal plants of interest include *Aspalathus linearis*, *Camellia sinensis*, *Moringa oleifera*, *Morinda officinale*, *Taraxacum officinale*, *Trichilia emetica*, *Terminalia sambesiaca*, *Peltophorum africanum*, *Ximenia caffra*, *Serenoa repens*, *Zingiber officinale*, *Eugenia jambolana*, and a combination of dandelion and fermented rooibos (CRS-10). According to the findings obtained from studies conducted on the evaluated medicinal plants, it can, therefore, be concluded that the medicinal plants maintain the antioxidant profile of Leydig cells under basal conditions and have protective or restorative effects following exposure to oxidative stress. The available data suggest that the protective role exhibited by the evaluated plants may be attributed to their antioxidant content. Additionally, the use of the optimal dosage or concentration of the extracts in the management of oxidative stress is of the utmost importance, and the measurement of their oxidation reduction potential is recommended.

## 1. Introduction

Leydig (interstitial) cells are endocrine cells that lie in the interstitial spaces located alongside the seminiferous tubules in the testes. These interstitial cells play a crucial role in steroidogenesis and spermatogenesis, which are important for male fertility, as they synthesize testosterone [1,2]. Before birth, testosterone secreted by fetal Leydig cells is important for the masculinization of the reproductive tract and external genitalia and the testicular descent into the scrotum, while those secreted after puberty are crucial for spermatogenesis, maintaining a mature male reproductive tract, and preserving fertility [2]. Leydig cells are vulnerable to extracellular sources of reactive oxygen species (ROS) as a result of their close proximity to the testicular macrophages [2,3]. The generation of ROS by activated macrophages not only affects invading microbes but also exposes nearby tissues and cells, in this case, the Leydig cells, to oxidative stress [3]. In addition, their presence between the interstitial tissue of blood vessels allows them to have a key role in the innate immune local response by releasing cytokines and chemokines, providing a defense mechanism against pathogens or potential damage to Leydig cells [4]. Consequently, an imbalance in antioxidant and ROS levels causes oxidative stress, which damages Leydig cells, and results in significant steroidogenic and spermatogenic dysfunction, contributing to male infertility [2].

Oxidative stress results due to an extreme production of oxygen-derived free radicals that counteract the activity of cellular antioxidants, leading to a disproportion between the levels of ROS and antioxidants. This stress has been confirmed to be among the underlying mechanisms that induce harm to Leydig cells through triggering lipid peroxidation, inducing apoptosis, damaging mitochondrial activity, and reducing testosterone production [5]. Low levels of testosterone produced by Leydig cells leads to impaired steroidogenesis, spermatogenesis, and male infertility [6,7].

Excessive production of ROS may suppress the cellular antioxidant capacity from endogenous (including both enzymatic and non-enzymatic) antioxidants, which include superoxide dismutase (SOD), catalase (CAT), glutathione peroxidase (GPx), and glutathione (GSH). However, exogenous antioxidants (e.g., tocopherols, polyphenols, retinol, and tetraterpenoids), obtained mainly from the diet, may provide secondary protective measures against free-radical-mediated damage [8]. There is an increased use of plants for medicinal purposes, owing to their health benefits, reduced side effects, affordability, accessibility, and diversity [9]. These plants contain natural antioxidants that may be utilized in regimens used to treat male infertility. The possible effects and cytotoxic levels of other medicinal herbs have not yet been confirmed [10]. However, there are a few studies that have demonstrated the beneficial effects of various medicinal plants on Leydig cell functions under basal conditions, including *Aspalathus linearis* (rooibos), *Camellia sinensis* (tea), *Moringa oleifera* (Sanjana, Horseradish tree, drumstick, Moringa), *Trichilia emetica* (Natal mahogany), *Terminalia sambesiaca* (cluster leaf), *Taraxacum officinale* (Dandelion), *Peltophorum africanum* (Rhodesian blackwood), *Oryza sativa L* (Asian rice)*,* and *Ximenia caffra* (sour plum) [9,10,11,12,13,14,15]. Additionally, the protective effects of *Morinda officinalis* (Ba-ji-tian), *Zingiber officinale* (common ginger; Zing), *Eugenia jambolana* (Black Plum, Jamun, Java Plum), *Serenoa repens* (Saw palmetto), and CRS-10 (a combination of *dandelion* and fermented rooibos) have been assessed following the induction of OS in Leydig cells [16,17,18,19]. This review paper outlines the impacts of oxidative stress on the cells and presents an overview of the effects of various medicinal plant extracts on Leydig cells, particularly the TM3 Leydig cells.

## 2. Leydig Cell Development and Function

The protein secreted by Sertoli cells, known as desert hedgehog (Dhh) protein, along with the platelet-derived growth factor alpha (PDGF-A), allow cells that express steroidogenic factor 1 (SF-1, NR5A1) to differentiate into fetal Leydig cells (FLCs) [20,21]. The FLCs function as key cells that ensure the testis successfully descends from the lumbar area into the low-temperature environment of the scrotum, by producing androgen and relaxin-like factors [22]. The formation and maturation of adult Leydig cells (ALCs) involve three different transitions of cells. Firstly, the progenitor Leydig cells (PLCs), which appear between days eleven and fourteen after birth within the testis, are the primary cell type in the lineage of Leydig cells. These cells express cytochrome P450 family 11 subfamily A member 1 (CYP11A1/CYP11A/P450scc), 3β-hydroxysteroid dehydrogenase (3β- HSD) cytochrome P450, family 17, subfamily A, member 1 (CYP17A), and a decreased type of the luteinizing hormone receptor (LH-R), and secrete mainly androsterone [22,23]. The PLCs grow over time and become oval, giving rise to the second transition of immature Leydig cells (ILCs) that are seen in rats’ testis between 28 and 35 days postpartum, and their multiplying capacity decreases. Immature Leydig cells contain a high number of smooth endoplasmic reticulum as compared to the PLCs and generate increased concentrations of 5α-reduced androgens and 5α-androstane-3α,17β-diol. Lastly, the matured Leydig cells develop during day 56 after the division of ILCs [22].

The Leydig cells’ primary function is testosterone synthesis, referred to as steroidogenesis. Synthesis of steroids includes transforming cholesterol into steroid hormones, in this case, testosterone, and requires steroidogenic enzymes or proteins for completion [24]. The steroidogenic enzymes are part of the cytochrome P450 group of oxidases and include numerous oxidative enzymes that consist of approximately 500 amino acids and 1 heme group (collectively known as cytochrome P450, pigment 450) [25]. The process of steroidogenesis is highly susceptible to ROS damage as this process generates ROS at basal conditions. In addition, ROS actions occur at the site where the process of steroidogenesis is being modulated by the P450 cytochrome enzymes [26].

**Figure 1:**
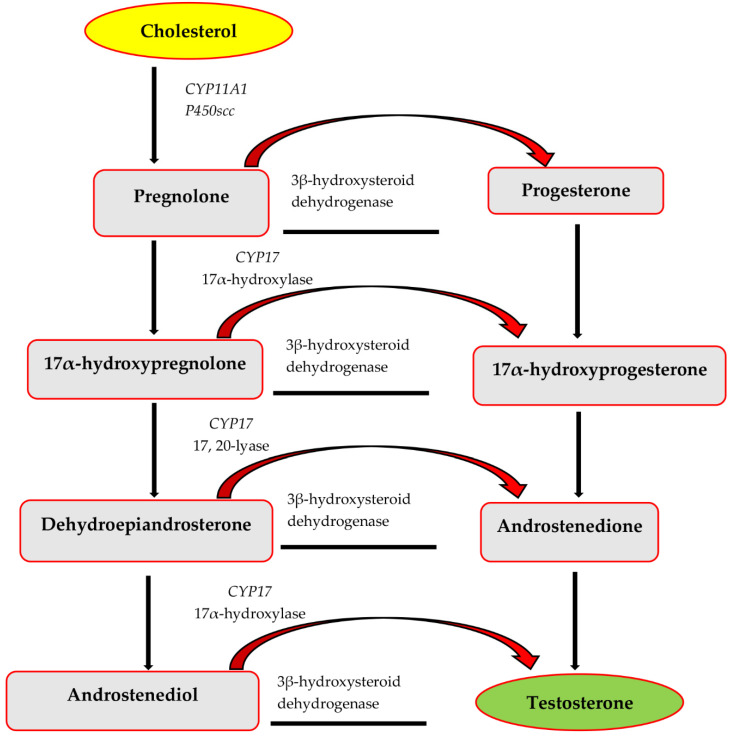
Biosynthesis of testosterone.

The synthesis of the steroid hormones occurs through the Δ4 or the Δ5 pathway, which is regulated by LH [27]. Lipid droplets and the plasma membrane initiate the biosynthesis of testosterone (Figure 1) through the mobilization of cholesterol. The primary and rate-limiting step of steroids synthesis involves transportation of cholesterol into the mitochondria under the influence of steroidogenic acute regulatory protein (StAR). In the Δ4 pathway (Figure 1), the C27 cytochrome P450 side-chain cleavage (CYP11A1) regulates the transformation of cholesterol into pregnenolone. Pregnenolone then diffuses into the smooth endoplasmic reticulum (SER), which is further transformed into progesterone, regulated by the activity of 3β-HSD. The 17-alpha (α)-hydroxylase (CYP17A1) transforms progesterone into 17-alpha (α)-hydroxy-progesterone, that is further converted into androst-4-ene-3,17-dione. Lastly, 17-beta hydroxysteroid dehydrogenase regulates the conversion of androst-4-ene-3,17-dione into testosterone hormone [7,11,28]. In the Δ5 pathway (Figure 1), 17-alpha (α)-hydroxylase enzyme modulates the transformation of pregnenolone into 17-alpha (α)-hydroxy-pregnenolone, which is further converted to androstenolone. Androstenolone is then catalyzed to androstenedione by 17β-HSD, and 3β-HSD enzyme regulates the transformation of androstenedione to testosterone [27,29]. The produced testosterone may either be transformed to 5α-dihydrotestosterone (catalyzed by 5-α-reductase) and/or 17β-estradiol (catalyzed by estrogen synthase) [30].

The conversion of cholesterol into testosterone via a cascade event with the aid of the steroidogenic enzymes is illustrated in Figure 1.

The biosynthesis of testosterone is regulated by the luteinizing hormone (LH, an anterior pituitary gonadotropic hormone), which binds to LH receptors located on Leydig cells’ membrane. LH binds to and activates G protein-coupled receptors, resulting in adenylyl cyclase activation, increased intracellular cAMP production, and cAMP-dependent protein phosphorylation via protein kinase A (PKA). Thus, LH is important in Leydig cells’ steroidogenesis as they maintain adequate levels of steroidogenic enzymes (trophic effect) as well as mobilize and transport cholesterol into the inner mitochondrial membrane (acute effect) [31]. LH’s trophic and acute effects are mediated by signaling pathways that begin with the generation of 3′,5′-cyclic adenosine monophosphate (cAMP synthesis). The acute stimulation of Leydig cells by LH results in cholesterol transfer into the mitochondria, which is mediated in part by the actions of the steroidogenic acute regulatory protein (StAR), translocator protein (18kDa; TSPO), and other transduceosome proteins [32]. The role of 3′,5′-cyclic adenosine monophosphate in steroidogenesis includes, first, promoting increased testosterone production by assembling cholesterol and conveying it to the steroidogenic cycle, during which StAR regulates cholesterol transportation from the mitochondrial outer membrane to the mitochondrial inner membrane [33]. The second role of cAMP involves the continuous occurrence of an intense stimulus to the expression of steroidogenic genes and enzymes, as well as the upregulation of their activities [34]. 

Proinflammatory mediators such as lipopolysaccharides (LPS), ROS, transforming growth factor beta (TGFβ), tumor necrosis factor (TNF), interferon-gamma (IFNγ), leukemia inhibitory factor (LIF), interleukin-1 (IL-1), interleukin 6 (IL-6), and nitrogen monoxide, act to inhibit the production of testosterone at several steroidogenic sites (including StAR, 3β-HSD, P450 c17, and 17β-HSD) (see the review in [34]). In addition, the uptake of cholesterol into Leydig cells is enhanced by autophagy, and an autophagy deficiency has been associated with the steroidogenic decline [35]. Hence, an autophagy dysfunction could inhibit testosterone production [36].

## 3. Sources of Reactive Oxygen Species (ROS)

Reactive oxygen species are extremely unstable and quickly react with other free radicals to produce additional oxygen-derived free radicals [37]. They have one or more unpaired electrons and include hydroxide (OH^−^), superoxide radical (O_2_^_^), nitrogen monoxide (NO), peroxyl radical (ROO), lipid peroxyl radical (LO_2_), and thiyl radical (RS^−^), and non-radical particles such as dioxygen (singlet) (^−1^O_2_), hydrogen peroxide (H_2_O_2_), chlorine oxoacid (ClHO/HClO), lipid peroxide (LO_2_H), and trioxygen (O_3_) [38,39]. The presence of the unpaired electron located in the exterior shell of these oxygen-containing free radicals makes them rapidly react and have a limited lifespan, hence acting at the site of their generation [40]. 

Production of ROS in Leydig cells occurs due to a number of membrane-bound organelles, such as cytosol, peroxisomes, the tubular network of membranes located inside the cytoplasm, known as the endoplasmic reticulum (ER), and mitochondria [3,34]. Leydig cells also contain steroidogenic cytochrome P450 enzymes that are involved in catalyzing the process of atoms losing their valence electron (oxidation), coming from metabolites of the steroidogenic cycle, as well as serve as a source of producing free radicals [41]. Moreover, LH stimulation can result in increased ROS production [41].

Leydig cells have an intracellular antioxidant defense system that prevents cellular damage by maintaining a balance between ROS and antioxidants [41]. However, where the Leydig cells’ repair mechanism is impaired due to extensive oxidative damage, the cells undergo programmed cell death, resulting in a reduced Leydig cell population and insufficient production of testosterone [42]. Mitochondrial harm resulting from oxidative damage is due to the development of hydroxide radical (OH^−^), which is a quite active and highly reactive oxidizing agent able to corrode lipids, carbohydrates, proteins, and DNA of Leydig cells [43].

- Mitochondrial respiratory chain

The mitochondria, which are considered as the initial area where ROS generation occurs, generate energy in the form of ATP through the process of utilizing energy obtained from the transfer of electrons in an electron transport system (oxidative phosphorylation) [44]. The process of the electron transport chain (ETC) causes several substrates to lose electrons (oxidize) and/or gain electrons (reduction) during a chemical reaction in the inner mitochondrial membrane, producing an electron flux that leads to the ATP production, and the molecular oxygen (O_2_) gains an electron to produce water (H_2_O) molecules. Under physiological conditions, some of the electrons (0.2–2%) directly leak out of the ETC and react with O_2_, forming O_2_^−^ and H_2_O_2_ via complexes I and III [44,45,46,47].

Production of ROS in complex I occurs when electrons are passed from NADH to ubiquinone/coenzyme Q_10_ (UbQ/CoQ_10_) via the flavin- (I_F_) and the UbQ-reducing (I_Q_) areas [48]. In the presence of NADH, complex I also generates O_2_•^−^ and is influenced by the binding of the inhibitor rotenone to the coenzyme quinone (CoQ)-binding region [49]. Production of the superoxide anion through NADH dehydrogenase (complex I) also results from the interaction of dioxygen (singlet) with the completely reduced riboflavin-5-phosphate (FMN). Hence, inhibition of complex I with rotenone enhances the generation of O_2_•^−^ as it reserves electrons in the FMN, which further generates O_2_•^−^ [50].

Complex III transfers electrons through the Q cycle [50] and produces high levels of superoxide anion via the interaction of O_2_ with semiquinone, attached to the Q_0_ region, in the presence of reduced coenzyme Q (CoQH_2_), as well as the inhibition of the Qi (inhibitor Q) site by antimycin [49,50]. The generated oxygen-containing free radicals are let out into the cell matrix and intermediate space (IMS), where they are translated to H_2_O_2_, being catalyzed by superoxide dismutase (SOD) [51]. Inhibition of antimycin A binding to the Q_i_ region has been reported to obstruct the passing of electrons from Q_0_ to Q_i_, leading to high electron leakage, followed by the synthesis of oxygen-containing free radicals at the Q_0_ region of the coenzyme Q reductase (complex III) [51,52]. It is also reported that the blockage of electron transfer can generate reverse electron transfer (RET), which produces high levels of ROS [51]. RET occurs when there is a reduction of the CoQ pool by the electron supply, which pushes back electrons from the ubiquinol (CoQH_2_) into NADH dehydrogenase.

b. Nicotinamide adenine dinucleotide phosphate (NADPH) oxidase-mediated ROS

As well as mitochondria, oxygen-derived free radicals are also synthesized by NADPH oxidase while converting NADPH to NADP^+^ [53]. NADPH oxidase is a multimeric complex comprising the NADPH oxidase enzymes family [54]. These membrane-bound proteins convey electrons over biological membranes to molecular O_2_, producing superoxide anion and, eventually, ROS [43]. Expression of NADPH oxidase enzymes occurs simultaneously; however, the number in which they are dispensed and expressed within tissues of the body is quite distinctive. For example, NADPH oxidase 1 is extremely expressed within the colon, NADPH oxidase 2 in neutrophils and monocytes (phagocytes), NADPH oxidase 3 is highly expressed within the internal ear (auris interna), NADPH oxidase 4 expression is high in the kidney and blood vessels, and NADPH oxidase 5 expression is extremely high within the lymphatic tissue and the testis [43]. In addition, NADPH oxidase 5 has been identified as the primary initiator for generation of oxygen-derived free radicals in the sperm of certain mammals [55].

Usually, ROS is produced by NADPH oxidase through the respiratory burst of phagocytic cells [44,56]. For instance, NOX4 forms H_2_O_2_ under physiological conditions, while the function of NADPH oxidase 1 and NADPH oxidase 2 is to produce O_2_•^−^, and NADPH oxidase 5 generates H_2_O_2_ when stimulated by calcium [57]. ROS production and release in an “oxidative burst” indicate the importance of phagocytic leukocyte cells in destroying microbes. This process requires high amounts of energy (ATP) that depend on glucose metabolism, and NADPH is the primary substrate for the respiratory burst [54].

c. Cytochrome P450 and Adrenodoxin reductase system

Steroidogenic tissues are at a higher risk of oxidative damage as they utilize molecular oxygen during the reaction of CYP450 with their substrates, which contributes to ROS production [58]. The cytochrome P450 enzyme categories include microsomal CYP450s, found with a complex network of tubular membranes (endoplasmic reticulum), and mitochondrial CYP450s, located within the internal mitochondrial membrane. The P450scc (CYP11A1) and P450c11β (CY11β1) found within the mitochondria are responsible for catalyzing crucial steps in generating steroid hormones [59]. Electron leakage occurs during steroidogenesis due to the transfer of cholesterol around the mitochondrial membrane, which is facilitated by StAR to the cleavage then further catalyzed by P450scc into pregnenolone, as well as the other steroidogenic steps catalyzed by the isoforms of P450 in the endoplasmic reticulum [59]. Evidence also indicates that StAR is vulnerable to extreme volumes of physiological and pathophysiological oxygen-derived free radicals [59]. A reduction in StAR protein’s expression level is depicted in response to increased ROS production [60,61,62]. 

Cytochrome P450 enzymes utilize electrons obtained from NADPH for the hydroxylation of substrates, which are transported via the electron transport chain by the activity of the adrenodoxin reductase (flavoprotein) and adrenodoxin [59,63], which may result in “uncoupling” or “leakage” of electrons during their transfer [26]. The uncoupling of electrons contributes to ROS production by converting oxygen to superoxide ion, which is protonated to form hydroxyl radicals or dismutation by the superoxide dismutase (SODs) to form H_2_O_2_ [59,64]. However, the rate at which electrons’ leakage occurs varies among the different P450 subtypes and steroid substrates. For instance, about 40% of the overall electron flow within the P450c11β system results in the production of ROS, as compared to 15% in P450scc [59]. Furthermore, Harskamp [65] reported that CYP1B1 and CYP1D1 showed increased rates of uncoupling in comparison to CYP1A1, CYP1C1, and CYP1C2. 

The cytochrome P450 enzymes, particularly CYP2E1, are shown to produce ROS and cause peroxidative alterations that lead to lipid peroxidation, whereby the end products of oxidative degradation of lipids (lipid peroxidation) are able to react with DNA and create oxidative DNA adducts [66]. Modified DNA may cause changes in the chromosomal and genetic structures (mutations) and cases of misreading in the replication process, and protein modification, especially amino acid cysteine, may consecutively harm proteins or result in downstream signaling in harmful mechanisms [64].

d. Leukocytes and inflammatory cytokines

Testicular spermatogenic and somatic cells produce several immunoregulatory and proinflammatory cytokines during basal and inflammatory circumstances [67]. In particular, Leydig cells and Sertoli cells produce cytokines, namely, interleukin-1 (IL-1), interleukin-6 (IL-6), and tumor necrotic factor (TNF) [67].

The presence of leukocytes within the testes interstitial spaces also provides innate immunity [68]. Macrophages, the major subsets of leukocytes, stimulate the inflammatory process by releasing cytokines such as interleukin-2 (IL-2), interleukin-4 (IL-4), interleukin-6 (IL-6), interleukin-8 (IL-8), and tumor necrotic factor-alpha (TNF-α) [68]. In addition, activating macrophages results in the production of proinflammatory cytokines (IL-1 and TNF), which are inhibitory to Leydig cells as they act as transcriptional repressors for steroidogenic genes’ expression, thereby preventing the process of steroidogenesis from occurring [34]. The macrophages also produce ROS, particularly H_2_O_2_, that results in the inhibition of StAR protein expression [34]. The increased levels of ROS and reactive nitrogen species when inflammation occurs expose Leydig cells to oxidative stress [34]. 

Leukocytes generate ROS by disintegrating pathogens via the activation of the myeloperoxidase system [69]. The staining of myeloperoxidase is vital for differentiating granulocytes such as polymorphonuclear leukocytes (neutrophils, eosinophils, and basophils) from sex cells, to identify what influences the high generation of oxygen-derived free radicals [70]. Leukocytes that stain positive for peroxidase appear brown in color, indicating their volume of generating excessive ROS production via phagocytosis. Furthermore, stimulated leukocytes enhance NADPH generation through the pentose phosphate pathway, permitting them to generate ROS that is one hundred times as much as the inactivated leukocytes [71].

**Figure 2:**
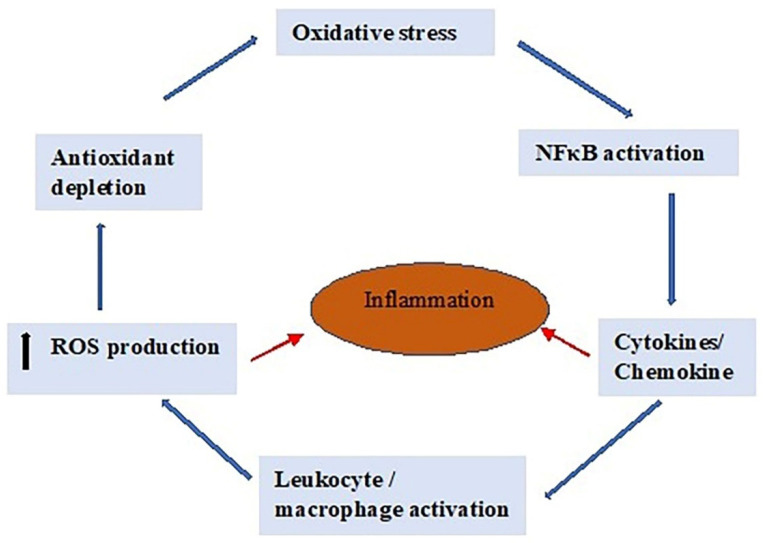
Perpetual cycle, showing the mechanisms of cytokine-dependent ROS production in inflammation. ROS, reactive oxygen species; NF-κB, nuclear factor kappa B.

ROS and proinflammatory cytokines are inextricably linked and perpetuate a cycle (Figure 2). ROS induces heat shock proteins, stimulating proinflammatory cytokines and cell-adhesion molecules’ (CAM) expression [72]. These outcomes lead to the stimulation of white blood cells (WBCs), producing ROS production by leukocytes and resident cells (e.g., macrophages, endothelial cells, and fibroblasts) [73]. Furthermore, proinflammatory cytokines stimulate NADPH enzymes within a short period of time, resulting in increased ROS generation through accumulation of these enzymes. Small volumes of hydrogen peroxide stimulate NF-κB, which is the overall transcription factor for a number of inflammatory cytokines, cell-adhesion molecules, and chemokines [74]. Prolonged exposure to H_2_O_2_ increases the response of functional NADPH oxidase isoforms to stimulus, and therefore, extreme generation of oxygen-containing free radicals continuously occurs in the cells. Since the synthesis of this form of free radicals exists as a basic constituent of stimulated immune cells, primary inflammation causes oxidative stress in impacted tissues. The net oxidative stress further exacerbates inflammation.

NF-κB, an inducible transcription factor, is activated in response to oxidative stress, and increases the production of inflammatory cytokines, chemokines, and adhesion molecules, which leads to further production of ROS by leukocytes and adjacent macrophages. This further exacerbates inflammation as a result of the imbalance between cellular antioxidant levels and ROS production.

Reproductive tract infections stimulate the release of proinflammatory cytokines (Figure 2), causing a decrease in the antioxidant defense system, and to reduce the damage against bacterial strains, excessive production of ROS occurs, which induces OS that harms Leydig cells [75]. Proinflammatory cytokines, IL-6 and IL-8, secreted by leukocytes act as regulators to activate pro-oxidants and the antioxidative system, which enhances the rapid production and release of ROS [76]. The response to inflammation by the Leydig cells results in extreme generation of interleukin-1 beta (β), cyclooxygenase-2 (COX-2), and nitric oxide synthase 2 (NOS-2), that are associated with reduced concentrations of antioxidant enzymes [77]. Inflammation is also linked with deteriorated mitochondrial membrane potential, impaired steroidogenesis, and Leydig cell apoptosis [78]. Proinflammatory cytokines (TNF-α, IL-1β, and IL-6) are involved in compromising testosterone production in TM3 Leydig cells [1,68].

## 4. Factors That Influence the Generation of Reactive Oxygen Species in Leydig Cells

Although ROS is required for male reproduction function, excessive generation of ROS could be detrimental. Various sources of ROS cause OS, which can be brought about by many factors that negatively impair the functions of the Leydig cells and have been classified as endogenous and exogenous factors. 

### 4.1. Endogenous Factors

- Aging

There is a decreased serum testosterone level with aging [79], as a result of decreased levels of LH, following changes in the hypothalamic–pituitary axis, as observed in most rat strains and human studies [80]. Furthermore, aging in men and Brown Norway rats is characterized by decreased serum testosterone and unaltered or increased LH levels, as well as a decrease in the ability of Leydig cells to produce testosterone in response to LH [28,81,82,83]. The decreased testosterone levels in the aging Leydig cells may be due to their unresponsiveness to LH, resulting in the reduction of cAMP production and protein kinase A activities [84]. LH receptor G protein-coupling deficiency may be responsible for the reduced cAMP production by the aged Leydig cells, as well as the increased degradation of cAMP [31,48]. Furthermore, LH stimulation causes arachidonic acid release in Leydig cells, and is metabolized by cellular lipoxygenases, epoxygenases, and cyclooxygenases (COX) [85,86]. The metabolites have been demonstrated to be capable of modifying steroidogenesis, in part via influencing the expression of the StAR protein [85]. For instance, COX-2 suppression resulted in dramatically enhanced T synthesis in MA-10 Leydig cells and rat primary Leydig cells, indicating that COX-2 can negatively affect steroidogenesis [85,87].

As cells age, there is often an imbalance between pro-oxidants and antioxidants, which can result in an altered redox state and an accumulation of oxidative damage to intracellular macromolecules, contributing to age-related functional impairments [88,89]. Aged rat Leydig cells produce significantly more reactive oxygen than young rat cells [90]. Leydig cells’ aging is associated with decreased expression of primary enzymatic and non-enzymatic antioxidants, such as Cu-Zn-SOD, Mn-SOD, glutathione peroxidase (GPX-1), microsomal glutathione S-transferase (MGST1), glutathione S-transferase (GSTM2), and glutathione (GSH), resulting in increased oxidative stress and lipid peroxidation (oxidative damage) [77,78,79]. Consequently, the age-related decreases in Leydig cell antioxidant activities, gene expression, and protein levels demonstrate that the loss of steroidogenic function that accompanies Leydig cell aging may result in part from an altered antioxidant defense system [91,92].

Exogenous testosterone replacement therapy, the stem cell target approach, and calorie restriction (CR) are some suggested interventions for the restoration of testosterone levels in aging males or to delay age-related changes in Leydig cells, although the former has potential risks [93,94,95,96]. CR has been proven to enhance OS and inflammatory parameters, especially with aging, as well as obesity [97].

b. Male reproductive tract infections

Male reproductive tract infections, such as epididymitis, sexually transmitted infections, testicular torsion, and inflammation of the testes [77], negatively affect Leydig cells and male reproduction due to the excessive concentrations of seminal leukocytes. Immune activation and inflammation, whether systemic or local, directly block the hypothalamic–pituitary–Leydig cell axis, interfere with spermatogenic cell growth, and may induce sperm antibody production [98].

A number of pathogenic microorganisms, such as bacteria, viruses, and parasites, may penetrate into the male reproductive tract and stimulate a sequence of inflammatory responses that impede male fertility [99]. Bacterial infections, such as *Chlamydia trachomatis* (*C. trachomatis*), *Neisseria gonorrhoeae* (*N. gonorrhoeae*), and *Brucella,* add to the number of male infertility cases [100,101,102,103]. Several pathogenic bacteria that infect the male reproductive tract have been identified, which include, *Escherichia coli* (*E. coli*), *Staphylococcus aureus* (*S. aureus*), *Ureaplasma urealyticum* (*U. urealyticum*), *C. trachomatis*, *N. gonorrhoeae*, *Streptococcus agalactia*, and *Staphylococcus saprophyticus* (*S. saprophyticus*). These identified pathogenic bacteria causes diseases such as chlamydiosis, gonorrhea, and ureaplasmosis, which ultimately lead to male reproductive tract infections [104]. For instance, *C. trachomatis* has the capability to invade and survive within cells of the host organism, and has been detected in Leydig cells, the urethra, epididymis, and prostate [105]. Even though *C. trachomatis* is unable to move by itself, it can, however, transmit infection to testicular cell populations, such as Leydig cells, sperm cells, and Sertoli cells, through its ability to hijack testicular macrophages [106]. Although the mechanism is not clearly understood, a possible explanation for the Leydig cells might be its location to adjacent testicular macrophages [34,100].

Inflammatory damage to the male genital tract causes increased ROS generation, while the pathogenic bacterial strains located in the reproductive tract further exacerbate the production of ROS, in association with the inflammatory response [107,108]. Specific pattern recognition receptors (PRRs) mediate the recognition of pathogen-associated molecular patterns by the immune systems, which results in an inflammatory response during infection [107]. Leydig cells have been shown to produce several PRRs, including the toll-like receptors (TLRs), with TLR3 and TLR4 being highly expressed [109]. The innate immune system of Leydig cells, which is mediated by TLR3, is thought to be responsible for the activation of NF-κB and IRF3, followed by the production of proinflammatory cytokines such as IL-6 and TNF-α, as well as IFN-α and -β [107]. TLR3 and TLR4 activation has been shown to suppress testosterone production in Leydig cells, which is mediated by the action of TLR-induced high levels of cytokines, TNF-α and IL-6, released in the cells [110]. TLRs mainly activate the NF-κB pathways, which results in inflammatory responses and the development of OS [107].

Testicular macrophages are the major sources of cytokines in the male gonads, although Leydig and Sertoli cells are reported to also produce them (IL-1 and IL-6) [107,111]. During inflammation, the cytokines regulate the migration of leucocytes into the tissue [107]. During inflammation, testicular macrophages play an important role in the stimulation of inflammatory agents, and ROS, which disrupts gonadal steroidogenesis, causes testosterone levels to fall, and interferes with normal spermatogenic activity [112]. TLR-induced activation of p38 MAPK and NF-κB in macrophages results in the production of inflammatory cytokines and the development of OS, which results in testicular OS and death [113]. Consequently, OS is responsible for the effect of inflammation-induced oxidative damage on the Leydig cells, including disruption of mitochondrial physiology, failure of the StAR to activate cholesterol transport into mitochondria, and inhibition of steroidogenesis [114]. An inflammatory stimulus activates macrophage function, triggering a cascade of events and secretions that interact with the HPG axis at all levels, inhibiting gonadotropin secretion and steroidogenesis, while stimulating the HPA axis. The inhibitory regulation of steroidogenesis in response to inflammation appears to include direct neural pathways from the CNS, and as a result, inflammation severely impairs the Leydig cells’ ability to make testosterone [98]. Although, hormones produced by the adrenal glands and testis exert feedback inhibitory effects on the inflammatory process, resulting in inflammation resolution and testicular testosterone production recovery [98].

Furthermore, OS can bring about inflammation by activating several signaling pathways [107]. For instance, ROS and H_2_O_2_ cause inflammation by activating the NF-κB [115]. Additionally, OS activates Nod-like receptor protein 3 (NLRP3) inflammasome, an oligomeric molecular complex that activates innate immune responses by generating proinflammatory cytokines (IL-1 and IL-8) [116,117,118].

### 4.2. Exogenous Sources of ROS

- Psychological stress

Psychological stress is a mental strain or an intense feeling that overworks the brain beyond the limit of its function [119]. Psychological stress most likely causes an endocrine imbalance and contributes to infertility [120]. In one study, psychological stress was evaluated among infertile couples using the hospital anxiety and depression score (HADS), and those with a high HADS (≥8) had lower serum testosterone, and higher FSH and LH, compared to those with a normal HADS [120]. The level of total testosterone in the blood revealed a strong negative connection with the HADS total and anxiety scores. This suggests that testosterone levels decrease when a person’s psychological stress level rises [120]. In comparison to individuals with normal HADS, those with a high HADS had a reduced sperm count, motility, and morphologically normal spermatozoon, indicating that psychological stress impairs sperm functions, and could result in male infertility [120].

Psychological stress suppresses male reproductive functions by directly influencing glucocorticoid action on Leydig cells [121]. Increased cortisol levels and apoptosis of Leydig cells, with decreased testosterone synthesis, were seen in stressed male rats [122]. The glucocorticoid-induced Leydig cell apoptosis is thought to be mediated by the glucocorticoid receptors [122]. Furthermore, synthesis of glucocorticoids via 11β-hydroxysteroid dehydrogenase (11β-HSD) prevents the function of steroidogenic enzymes, and as a result, impairs the process of steroid synthesis from occurring in the Leydig cells, for which the reduction in testosterone levels occurs without altering the LH levels [123].

Furthermore, acute stress suppressed the HPG axis by inhibiting GnRH secretion, consequently inhibiting the release of LH from the pituitary gland, while stimulating the HPA axis [124]. Stimulation of the HPA axis results in the excess production and release of corticotropin-releasing hormone (CRH) and corticotropin and β-endorphin (β-EP) by the hypothalamus and pituitary gland, respectively, causing an increased release of cortisol by the adrenal glands [125]. The elevated release of β-EP impairs the release of GnRH from the pituitary gland, consequently leading to reduced release of FSH and LH and inhibition of testosterone production by Leydig cells [126,127], thereby impairing spermatogenesis and male fertility.

b. Heat stress on gonads

For normal steroidogenesis and spermatogenesis to occur, the testes are situated within the scrotum to maintain an optimum temperature between 2 and 4 °C below the normal body temperature [128]. Fever, cryptorchidism, and modern lifestyle factors, including sauna, steam room, tight underwear, the use of a laptop placed on top of one’s thighs, sitting or driving for a long time, and the use of electric blankets, induces scrotal hyperthermia, resulting in male infertility [129]. Scrotal hyperthermia has been identified as one of the factors that contribute to male infertility, however its mechanism of action in inducing dysfunctional spermatogenesis and steroidogenesis is not clearly understood [130]. An increase in degenerative Leydig cells, a decreased number of testosterone-positive Leydig cells in the interstitial area, a dilated smooth endoplasmic reticulum, enlarged mitochondria, and vanished mitochondrial cristae following scrotal hyperthermia have all been seen [131], all of which indicate histopathological changes to Leydig cells. Scrotal hyperthermia significantly reduced the sperm count, motility, viability, and the serum testosterone level, as well as altered sperm morphology in animal models [132,133,134]. A significant decrease in the volume of the testis, seminiferous tubule, and interstitial tissue, as well as a decrease in the number of testicular cells, including primary spermatocytes, spermatogonia, Leydig cells, and round spermatids, was also noted following scrotal hyperthermia in mice [133]. The decrease in testosterone levels and the change in BTB produced by damage to Leydig cells and Sertoli cells in scrotal heat leads to testicular cell apoptosis and spermatogenesis disruption [135,136,137], leading to male fertility problems. Furthermore, heat stress also significantly reduced the level of expression of 3β-HSD, as well as steroidogenic enzymes, Cyp11a1 and Hsd3b1, and downregulated the expression of cytochrome P450 family 17 and StAR protein, important for the biosynthesis of testosterone in Leydig cells [138,139].

Hyperthermia elevates the production of ROS, lipid peroxidation, and the mitochondrial O_2_^•−^ level, while decreasing the intracellular antioxidant enzymes, such as GSH levels and SOD and GSH-Px activities, as well as mRNA levels [135,140,141,142]. Furthermore, gonadal hyperthermia increases NADPH oxidase activity, breakage of DNA strands, and secretion of cytochrome c by the mitochondria [141,143]. A study indicated high levels of H_2_O_2_ and lipid peroxidation, with decreased activities of SOD and catalase in rat testes following exposure to heat [144]. Another study demonstrated that heat stress also increased OS, lipid peroxidation, SOD activity, phospholipid-hydroperoxide glutathione peroxidase (PHGPx), superoxide dismutase 2 (SOD-2), hypoxia-inducible factor 1 subunit alpha mRNAs (HIF-1α mRNAs), programmed cell death, and androgen biosynthesis in mice testes [143]. In addition, testicular heat stress resulted in programmed cell death of Leydig cells and low biosynthesis of testosterone in adult rats [131], as well as negatively impacted the function of Sertoli cells, and the generation of androgen-binding protein (ABP), steroidogenesis, and spermatogenesis [143], which further highlights the detrimental effect of heat stress on male fertility. Antioxidant therapy is suggested to be effective and feasible for the treatment of heat stress, as antioxidants maintain cell homeostasis and increase cellular tolerance to high-temperature environments by inhibiting the formation of oxidative stress [138].

c. Environmental toxicants

Environmental toxicants generate an imbalance in pro-oxidant/antioxidant levels, which leads to the production of ROS, and the activation of extrinsic (Fas and FasL) and intrinsic (mitochondrial) apoptotic pathways that bring about apoptotic damage to the testis [145]. Many environmental toxicants or their metabolites act as a pseudo-substrate for cytochrome P450 enzymes in the steroidogenic pathway, resulting in enhanced ROS production [146]. These environmental toxicants have been found to disrupt steroidogenesis by interfering with one or more steps of the process [146]. Interference at any point in the sequence of steroidogenesis, from LH binding to its receptor through the steroidogenic reactions in the smooth endoplasmic reticulum, could disrupt Leydig cell steroidogenesis, leading to infertility [146]. Furthermore, environmental toxicants inhibit androgen receptor binding (AR binding), mainly by affecting the development during puberty and suppressing spermatogenesis. AR-binding toxicants can also increase LH levels, which results in Leydig cell hypertrophy, hyperplasia, and tumors [22].

Environmental toxicants include heavy metals, air pollution pesticides, and radiation [44,145,146]. Majority of the toxicants that are detrimental to Leydig cells’ function, formation, and steroidogenesis are classified as antiandrogenic and estrogenic molecules [147]. The antiandrogenic toxicants are further classified as class 1A, that induces direct effects on steroid synthesis or indirectly affect Leydig cell formation and LH secretion, thereby inducing apoptosis of Leydig cells, and class 1B antiandrogenic toxicants, that obstruct the stimulation of androgen receptor (AR). Estrogen toxicants, on the other hand, bind to estrogen receptor 1 (ESR1) and stimulate ER, elevating CYP19 release or preventing the metabolism of estrogen [147]. Examples of class 1A antiandrogenic toxicants include phthalates (diethylhexyl phythalate (DEHP), di-n-butyl phthalate (DBP), di-isononyl phthalate (DINP), and benzyl butyl phthalate (BBP)), organophosphates (dimethoate, parathion, trichlorfon), organochlorines (lindane, aldrin, heptachlor), herbicides (glyphosate molinate and atrazine), and heavy metals (arsenic, cadmium, lead, silver, and cobalt). Class 1B of androgenic toxicants are organochlorines (dichlorodi-phenyltrichloroethane (DDT) and dichlorodiphenyldichloroethylene (DDE)) and dicarboximide, and estrogen toxicants are plasticizers (bisphenol A), organochlorines (metaphor), and polychlorinated biphenyls [22].

Increased administration of phthalates disturbs the formation of Leydig cells and steroidogenesis [130,148]. Rats exposed to DEHP during pregnancy produced male rats with delayed fetal Leydig cell formation in developing testes and caused decreased testosterone synthesis [149,150]. Organophosphate suppressed steroidogenesis in mouse tumor Leydig cells via downregulation of StAR and CYP11A1, without inducing alterations in 3β-HSD1 [151]. Indane impairs testosterone synthesis via hCG-stimulated Leydig cells in a dose-dependent manner and has also been shown to reduce cAMP synthesis through the decrease of the LHGCR number [152,153]. On the other hand, the administration of aldrin and molinate (herbicide) was found to induce reduced circulating and intratesticular testosterone levels in Leydig cells of rats [154].

Bisphenol A (BPA), a monomer used in the production of plastics and other products, is a common environmental toxicant, that decreases plasma levels of testosterone and LH, cholesterol carrier protein, and steroidogenic enzymes, and the numbers of Leydig cells [155,156], and has been demonstrated to trigger apoptosis in Leydig and germ cells by upregulating Fas, FasL, and caspase-3 [157]. Low doses of BPA (0. 01 nmolL^−1^) reduced testosterone synthesis in rat Leydig cells through downregulation of CYP17A1 [158].

Polychlorinated biphenyls (PCBs; Aroclor 1254) are organochlorine chemicals that have 1–10 chlorine atoms bonded to biphenyl [22]. Exposure to high levels of PCBs in rats for a period of 30 days decreased the circulating testosterone level, the luteinizing hormone choriogonadotropin receptor (LHGCR) number, and expressions of CYP11A1 and 3β- and 17β-HSDs [159]. Additionally, PCBs inhibited basal and LH-stimulated testosterone production, as well as activities of steroidogenic enzymes (P450scc, 3β- and 17β-HSDs), enzymatic (GSH, GPx, SOD, CAT), and non-enzymatic (vitamins C and E) antioxidants in Leydig cells [160]. This suggests that PCBs inhibit steroidogenesis via attenuating the activities of cytochrome P450scc. The negative effects of PCBs on Leydig cells can be mitigated in part by treatment with the antioxidant vitamins C and E, implying that its effects are caused by ROS [161].

An intermediate used in manufacturing pesticides, 2-bromopropane (2-BP), decreased testosterone synthesis and impaired the antioxidant cellular defenses associated with DNA damage, with increased lipid peroxidation in primary cultures of Leydig cells [162].

Cadmium is a common environmental pollutant in many industrial processes and smoking, as it is a byproduct of other metals’ production, such as zinc, lead, or copper, and is mostly utilized in batteries, pigments, coatings and electroplating, and plastic stabilizers, among other uses [163]. Humans are exposed to cadmium through contamination in air, drinking water, food, and through smoking, and it has been shown to impair male fertility via the production of ROS in the testis [163,164]. Cadmium increased ROS production, while decreasing antioxidant enzymes such as GSH, CAT, SOD, GPx, and glutathione reductase, as well as upregulating the expression of anti-apoptotic genes (Bax and TNF-α) and downregulating the expression of a pro-apoptotic gene (Bcl2) in the testis of male rats, which brought about a decrease in cell proliferation [165,166]. Furthermore, cadmium exposure decreased the number of spermatogonia, Sertoli cells, and Leydig cells, and inhibited testosterone production in male rats [167]. Additionally, cadmium decreased testosterone production, StAR mRNA levels, and the activities of testicular 3β- and 17β-HSDs [168], implying that cadmium-induced ROS suppressed testicular steroidogenesis. Furthermore, cadmium chloride significantly reduced the viability of Leydig cells and testosterone levels, in contrast to the control group [169]. It also resulted in high ROS levels and low actions of antioxidant enzymes and steroidogenic enzymes (StAR mRNA, 3β-HSD1, and 17β-HSD3) [22,169].

Arsenic has also been identified as a reproductive toxicant that causes malformations and pollutes the air, soil, and water. It has been found that exposure to 14 mg/L of arsenic oxide for 30 days decreased the diameter of Leydig cells and altered spermatogenesis [170]. This heavy metal also prevents the role of enzymes involved in steroidogenesis and steroid biosynthesis (17β-HSD3 and 3β-HSD1), such as testosterone [22].

d. Electromagnetic radiation

A continuous exposure to electromagnetic radiation has been reported to play a role in the production of ROS in reproductive cells [76,140]. Exposure to sources of radiation (such as cell phones, wireless internet, environmental radiation, etc.) negatively affects the male reproductive hormonal axis by reducing the release of testosterone by the Leydig cells [171]. This radiation impacts LH levels but not FSH and prolactin (PRL) [172]. Exposure to electromagnetic waves has also been mentioned to negatively directly influence the pineal gland. As a result, it compromises the function of the sleeping hormone (N-acetyl-5-methoxytryptamine/melatonin) on the gonadotropin-releasing hormone (GnRH) pulse in the hypothalamus [129]. It is also hypothesized that heat waves activate oxidative stress production [173,174], which has been indicated to negatively affect the number of Leydig cells by inducing apoptosis [175].

e. Lifestyle factors

Lifestyle factors that significantly promote ROS generation and oxidative stress include cigarette smoking, alcohol abuse, narcotics, obesity, sedentary lifestyle, and diet [123,176].

According to a study conducted by Aboulmaouahib [177], smoking contributes more to infertility. Cigarette smoke has stable and unstable free radicals within its particles, including toxic, carcinogenic, and mutagenic substances [178]. As well as directly producing reactive oxygen radicals, smoking may also indirectly enhance OS by decreasing antioxidant defense systems [179,180]. Cigarette smoke enhanced the generation of O_2_•^−^ and H_2_O_2_, resulting in damaging the cells’ lipid membranes, proteins, enzymes, and deoxyribonucleic acid (DNA) [181]. Following a 13-week treatment, cigarette smoke reduced the population of Leydig cells in male rats and increased lipid peroxidation compared to the control [182]. Nicotine, found within cigarettes, downregulates the expression of nuclear receptor subfamily 5 group A member 1 (NR5A1), P450scc/CYP11A1, as well as 3-beta-hydroxysteroid dehydrogenase 1 (3β-HSD1) and steroidogenic factor 1 [183,184]. Additionally, nicotine (50 µM) prevented the growth of immature Leydig cells, induced apoptosis, and obstructed the mitochondrial membrane potential, as well as reduced the rate at which cellular enzymes responsible for steroidogenesis and steroid biosynthesis (steroidogenic enzymes) responded to the external stimulus for gene expression (downregulation) in vitro [184]. Furthermore, an in vivo study demonstrated that that Leydig cells exposed to nicotine showed reduced levels of LH and FSH, Leydig cell numbers, and steroidogenesis [184]. In addition, smoking can stimulate proinflammatory leukocytes, which elevate levels of ROS with the response of neutrophils, macrophages, and eosinophils to the inflammation resulting from smoking [185]. Nevertheless, the pathway of smoking cytotoxicity is complex due to the fact that tobacco smoke has different chemical compounds, such as nicotine (C_10_H_14_N_2_), tar, carbon monoxide (CO), and heavy metals [69].

Alcohol intake enhances the formation of ROS by boosting the activity of cytochrome P450 enzymes, altering specific metal levels (especially free iron or copper ions), and eventually, lowering antioxidant levels, thereby resulting in oxidative stress [186]. Chronic alcohol intake decreases blood testosterone, LH, and FSH levels by interfering with the neural and endocrine systems’ interconnections [187,188]. In another study, the chronic intake of alcohol resulted in low levels of testosterone and progesterone levels, whilst increasing LH, FSH, and prolactin levels [189]. Chronic alcohol intake also significantly increased thiobarbituric acid-reactive substances (TBARS), superoxide dismutase, and glutathione S-transferase, with a decrease in GSH, ascorbic acid, catalase, glutathione reductase, and glutathione peroxidase [187]. The detrimental effect of alcohol on the serum testosterone levels may be due to increased oxidative stress, which can harm Leydig and supporting Sertoli cells, as well as impair the HPG axis [187].

The use of numerous recreational drugs, such as cannabis, opioids, and narcotics, may impair male fertility, as they cause hypogonadism by interfering with the HPG axis [123,190]. Additionally, recreational drug use negatively affects Leydig cells’ functions [191]. For instance, cannabis sativa extract (marijuana) is the most commonly used illegal substance [192]. Cannabis primarily modulates reproductive functions via the endogenous endocannabinoid system (ECS), mainly anandamide and 2-arachidonoylglycerol, that act via the cannabinoid receptors CB1 and CB2 [123,192,193]. Acute or chronic administration of Δ^9^-tetrahydrocannabinol (THC) and cannabidiol (CBD), the major cannabinoids present in cannabis, had no effect on testosterone production or the spatial distribution of Leydig cells compared to controls in male rats [194]. Various studies on human males showed no difference in serum testosterone levels among marijuana users compared to non-marijuana users [195,196,197]. Likewise, a systematic review indicated a non-significant relationship between long-term marijuana usage and HPG axis hormones [198]. On the contrary, acute (˂10 joints per week) and chronic (>10 joints per week) consumption of cannabis may lower LH and testosterone levels [199]. Furthermore, THC significantly reduced testosterone levels in testis microsomes and murine Leydig cells [200,201]. Additionally, a reduction in the expression of LH receptors on the testis as well as the activity of 3-HSD was noted in mice fed with cannabis [202]. Cocaine is an alkaloid derived from the leaves of many species of the Erythroxylaceae family [192]. Chronic consumption of cocaine reduced the free testosterone concentration [203], but remained unchanged after intravenous low-dose injection [204], in men. In a rat model, chronic administration of cocaine did not cause any change in testosterone, FSH, and LH levels [205]. On the other hand, intraperitoneal injection of low-dose cocaine increased testosterone levels, while the LH level remained unchanged; however, high doses caused no change in testosterone levels [206]. Further studies are necessitated to understand the effect of the various recreational drugs on Leydig cells and the male reproductive functions, as well as their mechanisms of action.

The Western diet is characterized by energy-dense, refined, and nutritionally deficient foods, such as high-energy sugars, trans-fatty and hydrolyzed fatty acids, omega-6 polyunsaturated fatty acids, and processed foods, as well as a reduction in the intake of fruits and vegetables, omega-3 polyunsaturated fatty acids, important micronutrients, antioxidants, and phyto-compounds [207]. Obesity, caused by relative overnutrition and a sedentary lifestyle, has emerged as a serious public health concern in recent decades [208]. A high-fat diet (HFD) is the most common cause of obesity [208]. Obesity alters various components of HPG in men, lowering testosterone synthesis and thereby impairing sperm production [209,210,211]. In one study, HFD reduced the steroidogenic capacity of Leydig cells of rats and serum testosterone levels [208]. In another study, HFD decreased IL-1β levels and increased testosterone in mice treated at the immature stage (TIS) but had the opposite effect in mice treated at the mature stage (TMS). Furthermore, IL-1β reduced testosterone secretion by downregulating P450scc and P450c17 gene expression. In addition, HFD reduced the number of macrophages in the testis as well as the expression of inflammasome-related genes and proteins in mice TIS [208]. IL-1β, which is found in testicular macrophages and/or Leydig cells, is a proinflammatory cytokine that promotes the release of several cytokines/chemokines, including IL-6, IL-8, IL-10, IL-13, MCP-4, and TNF-α [212]. Excess adipose tissue increases insulin resistance and plays a significant role in the development of oxidative stress, affecting reproductive pathways and sperm function [213]. In addition, excess adipose tissue increases the activity of aromatase, an enzyme responsible for converting testosterone to estrogen, that consequently results in a decreased testosterone level and impairment of the spermatogenesis [214]. Taken together, modifications of the respective lifestyle factors that minimize ROS formation and oxidative stress may enhance the functioning of Leydig cells, thereby improving sperm function and male fertility.

## 5. The Effects of Oxidative Stress on Leydig Cell Functions

Oxidative stress arises due to the imbalance between the generation of oxygen-containing free radicals and cellular antioxidants, thus overpowering the scavenging capability of the intracellular antioxidant defense system. It results in Leydig cell lipid peroxidation, lipoprotein injury, misfolded proteins, DNA fragmentation, inhibition of steroidogenic enzymes, and contributes to male infertility [215].

Lipid peroxidation negatively affects the structure and integrity of Leydig cells by transforming the permeability of membranes, which results in defective membrane receptors, reduced membrane-bound enzyme activities, and high rigidity of the Leydig cells’ membrane, ultimately reducing the membrane’s fluidity [8]. Free radicals induce lipid peroxidation, which in turn activates peroxide-metabolizing enzymes. The activity of peroxide-metabolizing enzymes can be reduced by suppressing gonadotropins through one of the following: the testosterone- or the gonadotropin-releasing hormone antagonist treatment. However, gonadotropin suppression results in sex cell atrophy, which might elevate degradation of lipids within seminiferous tubules and simultaneously reduce the Leydig cell number [216].

Oxidative stress also decreases testosterone production from damaged Leydig cells or causes injury to parts of the endocrine system, such as the adenohypophysis [129]. Under normal conditions, mitochondrial respiration, catalytic reactions of the steroidogenic cytochrome P450 enzymes, and steroid synthesis produce ROS in high concentrations. The excessive production of ROS induces OS, which impairs steroid production and causes injury to the Leydig cell mitochondrial membrane [7]. Should toll-like receptors expressed in Leydig cells fail to activate, testosterone production may be impaired [217]. Ultimately, programmed cell death of Leydig cells results from acute toxic disruptions of Sertoli cells and disturbs the testes’ microvascular networks, affecting the secretion of testosterone. Furthermore, the dysfunction of seminiferous tubules corresponds with the reduced Leydig cell number [218]. Leydig cells have receptors for insulin-like growth factor binding protein I (IGF-I) and platelet-derived growth factor A (PDGF-A). An insufficiency of IGF-1 reduces the testes and lowers the levels of serum testosterone testes’ size, lowering serum testosterone levels [219], while the deficiency of PDGF-A leads to a continuous decrease in testes’ size, dysfunction of germ cell genes, which induces a complete altered spermatozoa development (spermatogenesis arrest), and the absolute absence of matured Leydig cells [218].

Furthermore, mitochondrial DNA (mtDNA) is susceptible to oxidative damage, even at low production levels of oxygen-containing free radicals, due to it being located near the sites of ROS generation. During mitochondrial respiration, several reactive species are produced, which could lead to mutations such as base substitutions, missense mutations, and deletions [68]. The mutations impair the mitochondria’s capability to perform their vast number of metabolic roles, such as synthesizing ATP via the process of electron transport-linked phosphorylation. The repair process of mtDNA decelerates due to continuous subjection to oxidative harm. Moreover, oxidative harm can increase the likelihood of mitochondrial permeability transition pores. The damage can, therefore, activate an apoptosis cascade that promotes Leydig cell apoptosis, resulting in fewer Leydig cells, and subsequently leading to insufficient testosterone levels [42], impairments of spermatogenesis, and the synthesis of immature spermatozoa, with a loss of sperm mobility, viability, and capacity for fertilization [220].

## 6. Mechanism of Action of Oxidative Stress on the Leydig Cell Functions

**Figure 3:**
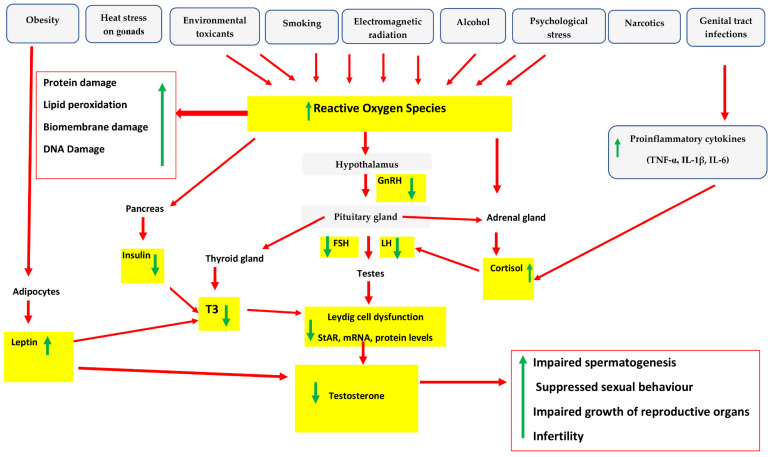
Impact of exogenous and endogenous sources of reactive oxygen species on hypothalamic–pituitary–adrenal, hypothalamic–pituitary–gonadal, and hypothalamic–pituitary–thyroid axes, and how they impair the Leydig cell functions.

Endogenous and exogenous factors that increase the levels of ROS in the male reproductive tract can cause an imbalance in the synthesis of oxidants and the scavenging ability of antioxidant enzymes, consequently resulting in OS, as demonstrated in Figure 3. Leydig cells produce ROS from several sources, including mitochondrial ETC, and mitochondrial and microsomal cytochrome P450 enzyme reactions [26]. OS has been shown to suppress Leydig cell steroidogenesis via decreased transcription of the StAR protein and the subsequent transport of cholesterol into the inner mitochondrial membrane for conversion into 17-hydroxypregnenalone [129]. ROS, specifically H_2_O_2_ derived from Leydig cells and testicular macrophages, cause mitochondrial dysfunction-mediated steroidogenesis to collapse via decreased transcription of steroidogenic enzymes, specifically P450 cholesterol side-chain cleavage (P450scc), 17-hydroxylase/17, 20-lyase (CYP17), and 3-hydroxysteroid dehydrogenase/54 isomerase (3-HSD) [221]. OS in Leydig cells is also associated with lower levels of cellular antioxidant enzymes, including SOD, GPx, and CAT, as well as the onset of apoptosis [222].

## 7. Impact of Oxidative Stress on Endocrine Axes

Elevated amounts of ROS may disrupt the endocrine axes (HPA, HPG, and HPT) and their crosstalk [223] (Figure 3). When ROS levels increase, cells respond by releasing stress hormones, i.e., 17-deoxycortisol (in animals) and cortisol/the stress hormone (in humans), which are activated by the HPA axis. These stress hormones signal to cross-communicate between HPA and HPG axes, reducing the release of LH by the adenohypophysis [224]. The excessive production of ROS activates HPA, which causes the hypothalamus to release CRH, that in turn activates secretion of adrenocorticotropic hormone (ACTH) by the frontal lobe of the hypophysis. ACTH activates the adrenal gland to secrete cortisol in response to OS, decreasing luteinizing hormone and follicle-stimulating hormone secretion from the adenohypophysis [178]. A miscommunication between the HPG and HPA axes further obstructs an increased concentration of LH receptor expression and enzymes involved in steroidogenesis and steroid biosynthesis [76]. The reduced LH release results in a failure to activate sufficient synthesis of testosterone by Leydig cells [225], while reduced FSH negatively affects androgen-binding protein (ABP) secretion by the Sertoli cells. This causes a net decrease in the flow of testosterone due to drastic OS [25], resulting in unregulated spermatogenesis, and may also suppress sexual behaviors [129]. Excess ROS production has also been demonstrated to impair LH signaling by mediating oxidation-sensitive MAPK pathways and inhibiting the mitochondrial cholesterol transport [226]. Additionally, FSH and human chorionic gonadotropin (hCG) activate the generation of ROS through cellular metabolism, negatively affecting the differentiation processes of germ cells. ROS production may be in response to LH, as Leydig cells exposed to LH had increased ROS levels and DNA damage [41].

Leydig cell dysfunction lowers triiodothyronine (T3) production, which reduces the general testosterone level [129]. Oxidative stress may also impact the hypothalamic–pituitary–thyroid (HPT) axis by decreasing the release of triiodothyronine (T3) and triiodothyronine (T4). A reduction in T3 lowers the levels of StAR, messenger ribonucleic acid (mRNA), and proteins in Leydig cells, and reduces the generation of testosterone [7]. In addition, severe OS causes insufficient concentrations of insulin to be secreted. In return, the thyroid gland fails to release T3, which results in an inadequate low circulating testosterone [25], and a failure to modulate the normal production of spermatozoa. One study indicated that insufficient levels of testosterone result in repressed sexual behavior in men, thus disturbing the endocrine and reproductive functions and contributing to male infertility [129]. Additionally, obesity results in excessive synthesis of oxygen-containing free radicals, which activates the activity of adipocytes to generate satiety hormone (leptin) and prevents the HPC axis activity. In addition, excessive ROS decreases insulin production from the pancreas, resulting in decreased testosterone production [178].

Consequently, the reduced testosterone fails to adequately regulate spermatogenesis, resulting in insufficient mature spermatozoa. It decreases sexual behavior as well as fails to maintain proper growth of accessory reproductive organs, which are important in sperm maturation [129], and may ultimately negatively affect male fertility.

Medicinal plants contain phytochemicals that have been shown to have protective effects on Leydig cells against ROS-induced damage due to their antioxidant activities [78,158,183]. The treatment must, however, be of an optimal dosage and duration, as excessive amounts of antioxidants can lead to reductive stress and cause adverse damage [227]. This implies that increased concentrations of antioxidants may be cytotoxic to Leydig cell function and obstruct neutralization or scavenging of free radicals. Hence, a balance between ROS production and antioxidant levels is essential for optimal functioning of Leydig cells [228]. Table 1 presents an overview of the effect of the selected medicinal plants on Leydig cells under basal and oxidative stress conditions. Table 1 shows that the respective medicinal plants enhanced the functions of Leydig cells following oxidative stress by increasing the antioxidant activities and the expression of steroidogenic enzymes for the synthesis of testosterone, while decreasing cell death, DNA damage, and lipid peroxidation, which may be attributed to their antioxidant properties. Therefore, medicinal plants combat oxidative stress through their antioxidant activities, thereby restoring the functions of the Leydig cells, steroidogenesis, and improving fertility outcomes.

## 8. Conclusions

To achieve Leydig cell protection against oxidative insults, there needs to be a balance between the redox state of oxygen-derived free radicals and the cellular protective capacity of antioxidants. Medicinal plants maintain the antioxidant profile of Leydig cells under basal conditions and have protective or restorative effects following exposure to oxidative stress. The available data suggest that the protective role exhibited by the evaluated plants may be attributed to their antioxidant content, as most of the medicinal plants have been reported to possess antioxidative effects against ROS-induced damage on Leydig cells. The use of the optimal dosage or concentration of the extracts in the management of oxidative stress is of the utmost importance, and the measurement of their oxidation reduction potential is recommended. Further studies are recommended to elaborate on the mechanisms of androgenic effects provided by these medicinal plants on Leydig cells.

## Figures and Tables

**Table 1:** Effects of various medicinal plants on Leydig cells.

Scientific and Common Names of Medicinal Plants	Subjects or Cell Types	Basal Conditions or Induced to OS	Intervention Period and Dosage	Methods/Techniques Used	Results	Conclusions/ Recommendations	Author(s) and Year
*Moringa oleifera* (Sanjana, Horseradish tree, drumstick, Moringa; MO)	TM3 Leydig cells	Basal conditions and hCG-stimulated conditions	24 h exposure to aqueous leaf extract of *MO* at concentrations of 0, 10, 50, 100, 250, 500, and 1000 µg/mL	MTT assay to assess viable cells. ELISA kit to determine testosterone level. Colorimetric lipid peroxidation assay kit to assess lipid peroxidation. Colorimetric assays to measure total antioxidant capacity, SOD, CAT, GSH, and lipid peroxidation.	Cell viability remained unchanged. A significant increase in levels of testosterone synthesis by 34% and 45% was observed under stimulatory conditions at 500 and 1000 µg/mL, respectively (*p* < 0.05). No significant change in CAT and SOD activities, total antioxidant capacity, nor lipid peroxidation was observed (*p* > 0.05). The level of GSH significantly increased at 250 µg/mL (*p* < 0.05).	MO possesses androgenic properties and it can be used for male infertility treatment in cases such as testosterone replacement therapy. More investigations on the mechanisms of the androgenic effects of this medicinal plant on Leydig cells were recommended, including cAMP and intracellular Ca^2+^. Further research on the protective effect of MO following the induction of OS is recommended.	[13]
*Aspalathus linearis* (rooibos) and *Camellia sinensis (tea*)	TM3 Leydig cells	Basal conditions and hCG-stimulated conditions	TM3 cells treated with *A. linearis* and/or *C. sinensis* for 24 h at 250–5000 µg/mL	MTT assay and ELISA kits to measure viable cells and testosterone production, respectively.	Testosterone production significantly reduced by 16.3–37.9% under stimulatory conditions (*p* < 0.05). Cell viability and morphology were maintained in TM3 Leydig cells treated with both plants at 250–1000 µg/mL concentrations, but cytotoxic to the cells at 5000 µg/mL (*p* ˂ 0.05).	Antiandrogenic property of *A. linearis* and *C. sinensis* was observed.	[12]
*Morinda officinalis* (Ba-ji-tian; MR)	TM3 Leydig cells	Basal conditions and H_2_O_2_-induced OS conditions	24 h exposure to *MR* concentrations of 5, 10, 50, 100, and 250 µg/mL	MTT assay to assess cell viability. Enzyme immunoassay kit to measure lipid peroxidation. Determination of CAT and SOD activities.	Generation of testosterone significantly elevated (43.5 pg/mL) and cell viability (*p* ˂ 0.001) enhanced activities of SOD (7.49 IU/mg protein) and CAT (74.6 IU/mg protein) (*p* ˂ 0.001), and reduced the amount of lipid peroxidation (1.75 nmol/mg protein) (*p* ˂ 0.05).	MR extract protected TM3 cells against OS induced using H_2_O_2_.	[16]
*Oryza sativa L.* (Asian rice; RBE)	TM3 Leydig cells	Basal conditions	24 h treatment with 0, 1, 10, 25, and 50 µg/mL	MTT assay qRT-PCR Western blotting ELISA kit	No effect on cell viability. Increased mRNA and protein levels of steroidogenic enzymes, including StAR, CYP11A1, and CYP17A1	RBE modulate steroidogenesis via the increased levels of steroidogenic enzymes.	[11]
*Taraxacum officinale* (Dandelion)	Mouse Leydig cells	Basal conditions	24 h treatment with 0, 1, 10, 25, and 50 µg/mL	MTT assay qRT-PCR Western blotting ELISA	No change in cell viability. *T. officinale* significantly increased mRNA and steroidogenic enzyme levels (cholesterol side-chain cleavage enzyme and StAR) and testosterone generation.	Steroidogenic property of *T. officinale* was demonstrated by the increased amounts of enzymes responsible for the process of steroidogenesis and steroid biosynthesis. More investigations should be conducted to assess the possibility of this plant extract in modulating biosynthesis of steroids and improve late-onset hypogonadism.	[9]
*Peltophorum africanum Sond* (Rhodesian blackwood; *africanum*), *Trichilia emetica Vahl* (Natal mahogany; *T. emetica*), *Terminalia sambesiaca* (clusterleaf; *T. sambesiaca*), and *Ximenia caffra* (sourplum; *X. caffra*)	TM3 Leydig cells	Basal conditions and stimulated with anti-hCG	24 h treatment with 0.1, 0.25, 0.5, 0.75, and 1 mg/mL	MTT assay ELISA kit	Cell viability was maintained enhanced. Testosterone level significantly increased at different concentrations of the various plant extracts (*p* < 0.05).	These medicinal herbs show antioxidative and androgenic properties, and the possible utilization of these herbs is recommended in the treatment of male infertility.	[10]
*Serenoa repens* (saw palmetto; SP)	TM3 Leydig cells	Basal and H_2_O_2_-induced conditions	1 h exposure to H_2_O_2_ (400 µM) and treatment with 10, 25, 50, 100, and 200 µg/mL of SP	RNeasy Mini kit for extraction of total RNA. iScript^TM^ cDNA synthesis kit for the synthesis of cDNA RT-PCR.	Testosterone biosynthesis was induced by stimulating expression of meRNA genes encoding 17,20-desmolase and 3β-hydroxysteroid dehydrogenase 4.	SP supplementation improved andropause symptoms via the direct or indirect modulation of testosterone synthesis in the Leydig cells.	[19]
Combination of dandelion extract and fermented rooibos extract (CRS-10)	TM3 Leydig cells	Basal and H_2_O_2_-induced conditions	2 h treatment with either 10 or 50 µg/mL of CRS-10, ED, or ER, before exposing cells to 40 µM of H_2_O_2_ for another 2 h	Alamar Blue assay	CRS-10 increased Leydig cell viability and production of testosterone (*p* < 0.05).	It was then concluded that CRS-10 is effective and safe for usage for the treatment of andropause symptoms.	[17]
*Zingiber officinale* (common ginger; Zing)	TM3 Leydig cells	Basal and ZEA-induced OS conditions	24 h treatment with 25 µM of ZEA and 25 µM of Zing	CCK-8 assay kit for cell viability. qRT-PCR technique. Western blotting. Bradford protein assay. ELISA kit. JC-1 staining assay for mitochondrial membrane potential.	Zing protected TM3 Leydig cells against ZEA-induced OS, via the increased cell viability, testosterone production, mitochondrial membrane potential, and levels of steroidogenic genes and proteins.	Zing possesses a protective effect against ZEA’s toxic effects on TM3 Leydig cells.	[18]
*Eugenia jambolana* (Java plum; EJE)	Rat Leydig cell line	Normal and OS-induced conditions	4 h treatment with 100 µg/mL	Viable cells were assessed using the trypan blue dye exclusion test. Assessment of lipid peroxidation, SOD, CAT, and GST activities, and GSH levels. Commercially available kit was used to assess TAC. Protein levels were determined using the Bradford protein assay.	Cell viability significantly increased (*p* < 0.05). Glutathione level and total antioxidant capacity significantly improved (*p* < 0.05). The programmed cell death influenced by H_2_O_2_ showed a significant decrease, which was observed by the downregulation of apopain (CPP32) and poly-ADP-ribose polymerase (PARP).	EJE can neutralize ROS as well as anti-apoptotic potency against the adverse impacts of H_2_O_2_ on Leydig cells in vitro.	[229]

Abbreviations: hCG, human chorionic gonadotropin; TAC, total antioxidant capacity; CAT, catalase; OS, oxidative stress; SOD, superoxide dismutase; GSH, glutathione; GST, glutathione S-transferases; ZEA, Zearalenone; Zing, Zingerone.

## Data Availability

No new data were created or analyzed in this study. Data sharing is not applicable to this article.
